# Prolyl Isomerase Pin1 Protects Mice from Endotoxin Shock

**DOI:** 10.1371/journal.pone.0014656

**Published:** 2011-02-04

**Authors:** Hirotada Akiyama, Takuma Misawa, Masao Ono, Chiyoko Uchida, Takafumi Uchida

**Affiliations:** 1 Molecular Enzymology, Department of Molecular Cell Science, Graduate School of Agricultural Science, Tohoku University, Sendai, Japan; 2 Department of Pathology, Graduate School of Medicine, Tohoku University, Sendai, Miyagi, Japan; 3 Center for Interdisciplinary Research, Tohoku University, Sendai, Japan; New York University, United States of America

## Abstract

**Background:**

Prolyl isomerase Pin1 may be involved in innate immunity against microbial infection, but the mechanism how Pin1 controls the innate immunity is poorly understood.

**Methodology/Principal Findings:**

Injection of lipopolysaccharide (LPS) into the mice induces inflammatory pulmonary disorder and sometimes the serious damages lead to death. Comparing to the wild-type (WT) mice, the Pin1^−/−^ mice showed more serious damages in lung and the lower survival rate after the LPS injection. We compared the levels of typical inflammatory cytokines. Pin1^−/−^ mice overreacted to the LPS injection to produce inflammatory cytokines, especially IL-6 more than WT mice. We showed that Pin1 binds phosphorylated PU.1 and they localize together in a nucleus. These results suggest that Pin1 controls the transcriptional activity of PU.1 and suppresses overreaction of macrophage that causes serious damages in lung.

**Conclusions/Significance:**

Pin1 may protect the mice from serious inflammation by LPS injection by attenuating the increase of *IL-6* transcription of the mouse macrophages.

## Introduction

The peptidyl prolyl *cis/trans* isomerase Pin1 specifically isomerizes phosphorylated serine or threonine residues preceding proline in certain proteins [1]. It has been reported that Pin1 regulates the functions of transcription factors, such as NFAT [2], STAT3 [3], and NF-κB [4] that play important roles in the immune system.

Pin1 regulates innate immunity that is an essential component of the immune system for host defense. The most important characteristic of innate immunity is the recognition of pathogens through Toll-like receptor (TLR). Thirteen TLRs have been identified so far. Each TLR specifically recognizes structural components of pathogens and triggers the innate immune response [5]. Lipopolysaccharide (LPS) is an outer membrane component of Gram-negative bacteria. It is specifically recognized by TLR4 [6]. Massive activation of innate immunity caused by LPS leads to excess production of cytokines and other molecules, and development of septic shock or endotoxin shock syndrome, a fatal syndrome that is characterized by fever, hypotension, disseminated intravascular coagulation, and multiple organ failure [7]. Pin1 affects LPS signal. Pin1 regulates degradation of inducible nitric oxide synthase and inhibits the production of LPS-induced nitric oxide in murine aortic endothelial cells (MAEC) [8]. Pin1 prevents the production of prostaglandin E2 in MAEC by regulating the degradation of cyclooxygenase-2 induced by LPS [9]. These results suggest that Pin1 weaken LPS signal and corresponding inflammation.

In this study, we compared LPS-induced inflammatory damage between WT and Pin1^−/−^ mice [10], and found that Pin1 plays a key role in suppressing LPS-induced PU.1 activation and protects mice from serious inflammation.

## Results

### Pin1 suppressed LPS-induced inflammation

**Figure 1:**
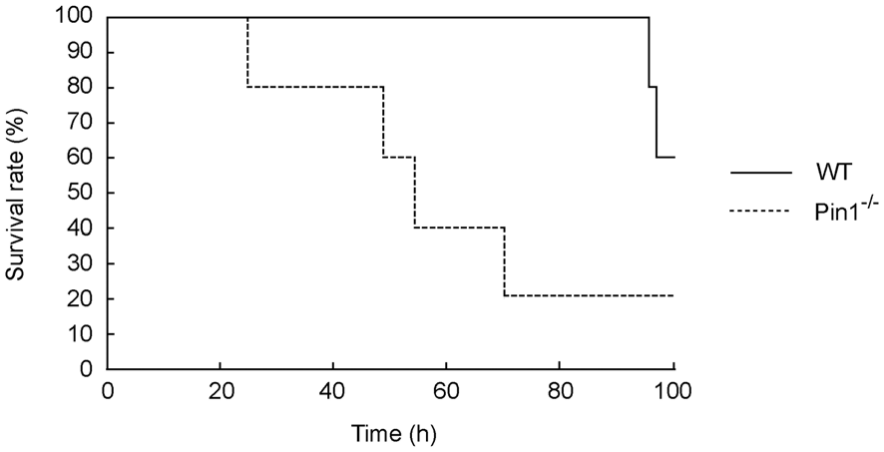
LPS induced more serious damage in Pin1^−/−^ mice. 15–20-week-old Pin1 WT (n = 5) and Pin1^−/−^ (n = 5) mice were intraperitoneally injected with LPS at 10 µg/g body weight or PBS and their survival rates was monitored.

**Figure 2:**
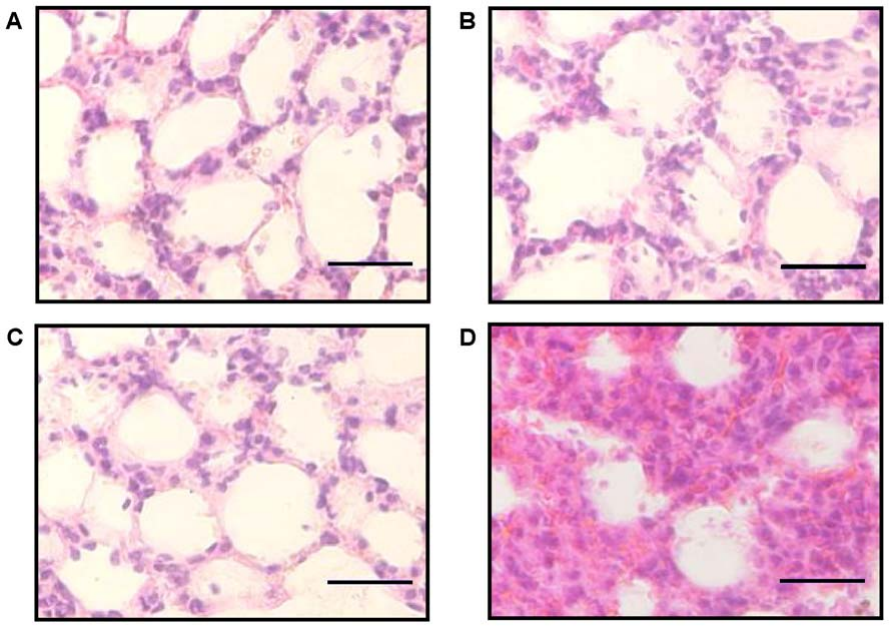
Histopathology of the lungs of WT and Pin1^−/−^ mice after LPS injection. Representative histological sections with hematoxylin and eosin staining of lungs from surviving mice that were sacrificed 100 h after LPS injection intrapenetoreally. The left panels show the control lungs of WT (A) and Pin1^−/−^ mice (C), and the right panels show the lungs of WT (B) and Pin1^−/−^ mice (D) that were injected with LPS for 100 h. Scale bars, 30 µm. control; n = 5 per genotype, LPS-injected; n = 5 per genotype.

To determine whether Pin1 plays a critical role in response to inflammatory signals, 15–20 week old WT and Pin1^−/−^ mice were injected with LPS at 10 µg/g body weight intrapenitoneally and their survival rates were monitored. Injection of LPS into the Pin1^−/−^mice induced trepidation, lameness, and eye mucus. WT mice showed the same symptoms as Pin1^−/−^ mice, but they were less severely affected and survived significantly longer than Pin1^−/−^ mice (Figure 1). The median survival time of WT mice was 98.7±0.84 h and that of Pin1^−/−^ mice was 59.8±12.4 h after LPS injection (Table S1). Inflammation caused by LPS leads to the development of multiple organ failures in vivo [7]. WT and Pin1^−/−^ mice were sacrificed 50–100 h after LPS injection, and the histopathology of lung, liver, kidney, and spleen were investigated. As a result, we found that LPS injection caused excessive lung injury in Pin1^−/−^ mice than WT mice (Figure 2). We measured the levels of several inflammation- related cytokines, such as IL-12p70, TNFα, IFNγ, MCFP-1, IL-10 and IL-6 in serum of these mice at 24 hours after LPS injection by EIA. Among these cytokines, TNFα and IL-6 increased the most. TNFα was increased 2.5- and 3-folds and IL-6 was increased 3- and 10-folds in WT and Pin1^−/−^ mice respectively. These results indicate that Pin1 may protect mice from serious inflammation by regulating expression of IL-6 after LPS-injection.

### Pin1 selectively attenuated LPS-induced IL-6 transcription

**Figure 3:**
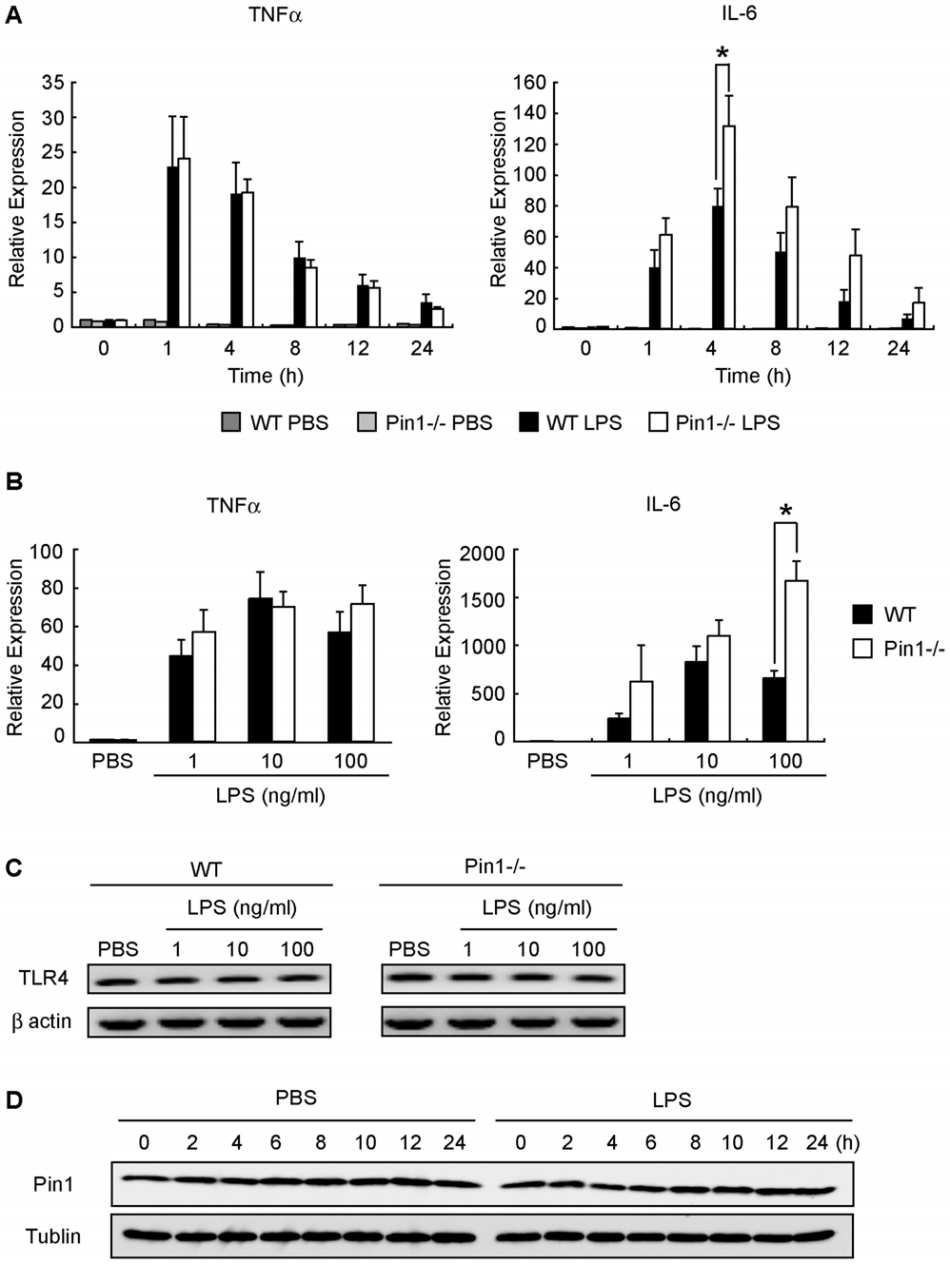
Comparison of TNFα, IL-6 and TLR4 mRNA and Pin1 protein in the macrophages of WT and Pin1^−/−^ mice after LPS injection. (A) Peritoneal macrophages from WT and Pin1^−/−^ mice were stimulated with 100ng/ml LPS for the indicated periods. Total RNA was extracted and then subjected to quantitative real-time PCR analysis using primers specific for *TNFα* and *IL-6.* (B) Peritoneal macrophages from WT and Pin1^−/−^ mice were stimulated with various concentrations of LPS for 4 h.Total RNA was extracted and then subjected to quantitative real-time PCR analysis using primers specific for *TNFα* and *IL-6.* (C) Peritoneal macrophages from WT and Pin1^−/−^ mice were stimulated with various concentrations of LPS for 4 h.Total RNA was extracted and then subjected to PCR analysis using primers specific for *TLR4.* (D) Peritoneal macrophages from WT mice were stimulated with 100ng/ml LPS for the indicated periods. At indicated time points, cell lysates were prepared and subjected to Western blotting analysis using anti-Pin1 and anti-tubulin as a control. mRNA levels were normalized to that of β-actin and then normalized to the relative mRNA level of WT at time 0 h or mRNA level of WT stimulated with PBS. Results are shown as means±SEM for 3 independent sets of experiments. Asterisk* denotes significant difference (p<0.05).

Macrophages produce inflammatory cytokines, TNFα and IL-6 in response to LPS stimulation, which causes serious damage in mice [11]. The TNFα mRNA levels of the macrophages in the WT and Pin1^−/−^ mice after LPS-injection were similar. On the other hands, the IL-6 mRNA level of the macrophages in the Pin1^−/−^ mice was higher than that of WT mice after LPS-injection. The difference was the most significant at 4 h after LPS stimulation (Figure 3A). The TNFα mRNA levels were increased similarly in WT and Pin1^−/−^ mice at any doses of LPS-injection, although the IL-6 mRNA level was always higher in the Pin1^−/−^ mice than in the WT mice (Figure 3B). The transcription level of TLR4 of Pin1^−/−^ macrophages was similar to that of WT (Figure 3C) and the expression level of Pin1 protein in WT macrophages was not changed by LPS stimulation (Figure 3D). From these results, we hypothesized that Pin1 constantly acts at downstream of TLR4 and inhibits the LPS-induced IL-6 transcription.

### Affect of Pin1 on LPS-induced IL-6 transcription in RAW264.7

**Figure 4:**
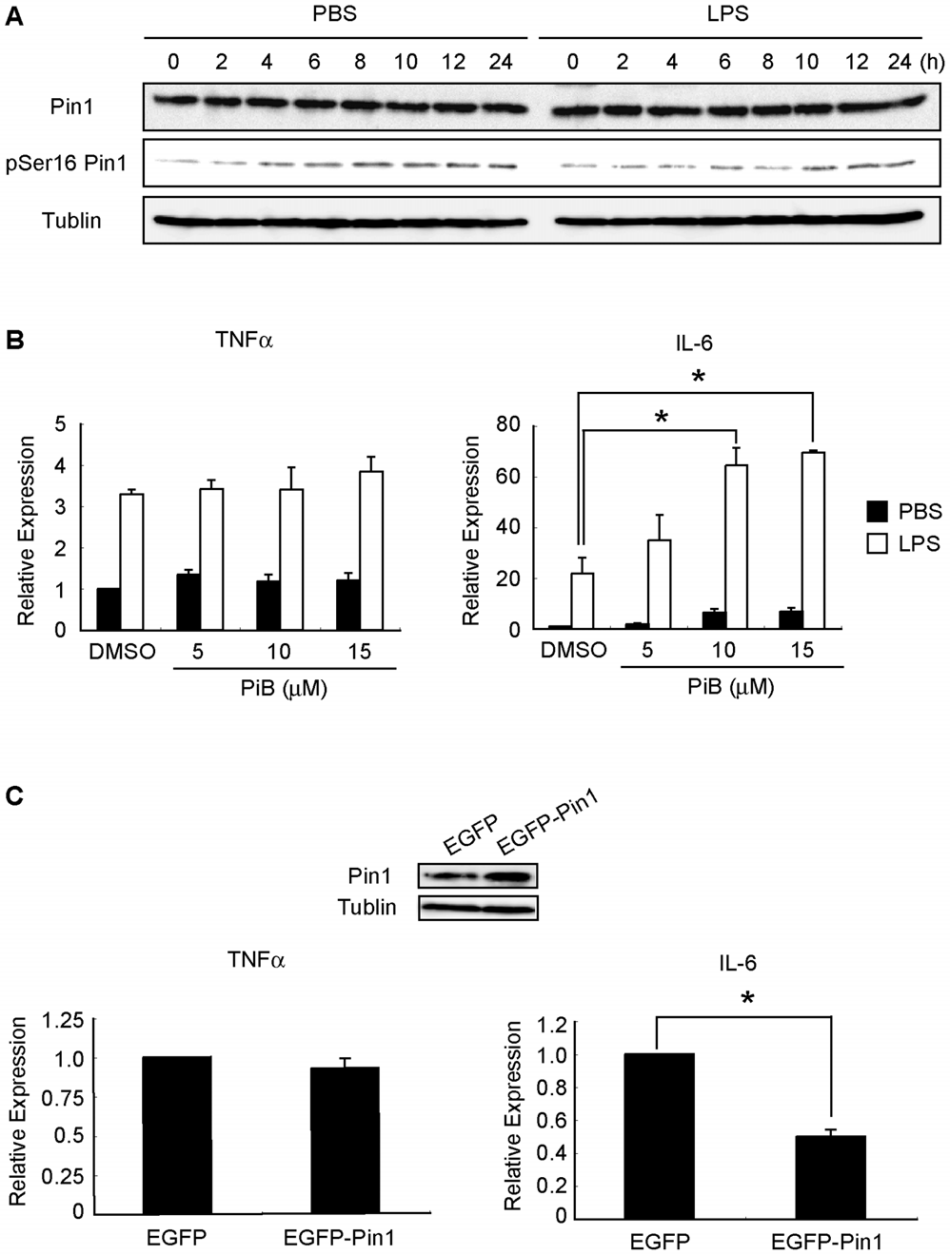
Function of Pin1 in RAW264.7 stimulated with LPS. (A) RAW264.7 was stimulated with 100ng/ml LPS for the indicated periods. At indicated time points, cell lysates were prepared and subjected to Western blotting analysis using anti-pSer16 Pin1 and anti-tublin as a control. (B) RAW264.7 was pretreated with Pin1-specific inhibitor, PiB and stimulated with 100ng/ml LPS for 4 h.Total RNA was extracted and then subjected to quantitative real-time PCR analysis using primers specific for *TNFα* and *IL-6.* (C) Pin1 overexpressed RAW264.7 was stimulated with 100ng/ml LPS for 4 h.Total RNA was extracted and then subjected to quantitative real-time PCR analysis using primers specific for *TNFα* and *IL-6.* mRNA levels were standarized by β-actin. Results were shown as means±SEM for 3 independent sets of experiments. Asterisk* denotes significant difference (p<0.05).

We next analyzed the effect of Pin1 in LPS-signal in mouse macrophage cell line RAW264.7. It has been reported previously that phoshorylation of Ser16 of Pin1 impairs the function of Pin1 [12]. We thought if Pin1 is activated at the downstream of TLR4 by LPS treatment, the amount of pSer16-Pin1 would be decreased. The pSer16-Pin1 level in RAW264.7- treated with LPS was lower than the control between 4 h–10 h after the treatment (Figure 4A). These results suggest that active type of Pin1 is increased by LPS stimulation. We next examined whether LPS induces the mRNA level of IL-6 in the RAW264.7- pretreated with Pin1-specific inhibitor PiB [13]. Although the TNFα mRNA level was not affected by the PiB treatment, the transcription level of IL-6 was significantly elevated in the cells- pretreated with PiB (Figure 4B). Additionally, over-expression of Pin1 in RAW264.7 results in decrease of LPS-induced IL-6 transcription, but not TNFα (Figure 4C). These results indicate that Pin1 is activated by LPS stimulation and selectively suppresses LPS-induced IL-6 transcription.

### Interaction of Pin1 with PU.1

**Figure 5:**
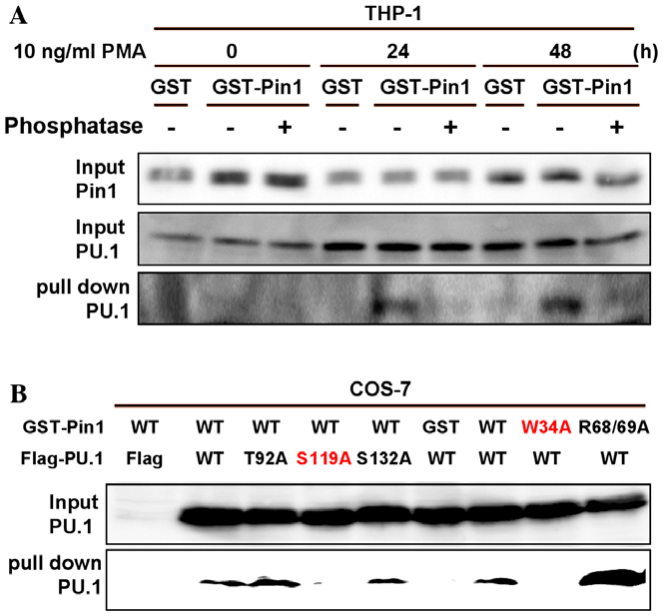
Pull down assay of PU.1 by Pin1. (A) The human macrophage cell line THP-1 treated with or without 10 ng/ml PMA were lysed with Lysis buffer. GST-Pin1and GST beads were incubated with the cell lysates treated with phosphatase previously or not. (B) Wild-type Pin1, Pin1 mutants in which Trp34 at the WW domain was mutated to Ala34 (Pin1_W34A_), and Pin1 in which Arg68 and Arg69 at the PPIase domain mutated to Ala68 and Ala69 (Pin1_R68/69A_) produced as GST-fusion proteins were bound to the glutathione-beads. PU.1 in which Thr92 or Ser119 were mutated to Ala were prepared and used instead of wild type of Pin1.

Stimulation of a human acute monocytic leukemia cell line, THP-1 with PMA increased the PU.1 that bound Pin1-beads, but phosphatase treatment decreased the amount of PU.1 (Figure 5a). The sites where Pin1 binds were mutated and examined if Pin1 bound the sites or not. As shown in Figure 5b, Pin1 did not bind S119A but bound T92A and S132A. These results showed that PU.1 bound S119-Pro site specifically. Although whole Pin1 and PPIase domain mutant, Pin1_R68/69A_ bound PU.1, WW domain mutant, Pin1_W34A_ did not bind PU.1.

### Colocalization of Pin1 and PU.1

**Figure 6:**
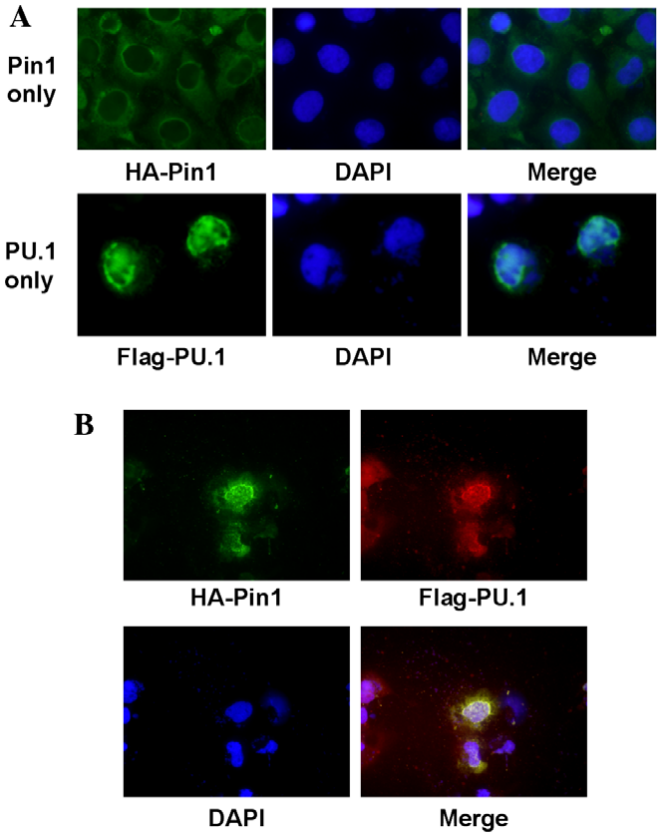
Immunofluorescent Cell Staining. COS7 cells were transfected with (A) *HA-Pin1* or *Flag-PU.1* and (B) *HA-Pin1* and *Flag-PU.1* together. After 48 h, cells were fixed with 4% paraformaldehyde, treated with HA probe and FLAG M2 antibodies and Alexa Fluor 488- and 595- conjugated secondary antibodies, and DAPI staining for nucleus. These cells were observed under fluorescence microscope (Biozero 8000, KEYENCE, Japan).

Transfection of COS7 cells with either Pin1 or PU.1 showed that Pin1 and PU.1 localized in cytosol and nucleus respectively (Figure 6a). But transfection of the same cell with both cDNAs together made both proteins localize in a nucleus (Figure 6b). The results suggest that Pin1 binds PU.1 in a cell and moves into a nucleus together.

## Discussion

In this report, we have shown that Pin1 selectively inhibits LPS-induced IL-6 transcription at the downstream of TLR4 in macrophages. However, it is still unknown how Pin1 specifically suppresses *IL-6* transcription after the LPS-stimulation. It has been reported that IκBNS that inhibits IL-6 production selectively in macrophages is induced by IL-10 and LPS [14]. Pin1 enhances the activity of transcription factors, CREB and STAT3 in several cell types, which increase IL-10 production in macrophages [15], [16]. Taken together, we assume that Pin1 may up-regulate the production of IL-10 by accelerating the transcription activity of CREB/STAT3 and elevates the level of IL-6 specific IκBNS inhibitor in macrophages in response to LPS stimulation.

We have also found that monocyte chemotactic protein-1 (MCP-1) was upregulated in Pin1^−/−^ mice and inhibition of Pin1 by PiB or juglone results in upregulation of chemotactic activity of macrophages (data no shown). These results indicate that Pin1 may play a role in regulating chemotactic activity of macrophages and prevents the expansion of inflammation to the entire body.

These results suggest that Pin1 controls macrophage maturation. We found that Pin1 binds phosphorylated Ser119 –Pro in PU.1 at the WW domain and the complex localizes in a nucleus. Taken together, we speculate that Pin1 regulates the transcriptional activity of PU.1 in a nucleus and suppress the excessive activation of macrophage.

In this report, we have shown the Pin1's new function in innate immune system. We do not think IL-6 up-regulation is only the cause of serious inflammation and immediate death of Pin1^−/−^ mice. Further study that discloses the precise molecule mechanisms by which Pin1 selectively inhibits TLR-dependent genes will provide basis for the development of new therapeutic strategies to a variety of inflammatory diseases.

## Materials and Methods

### Animal study

Our study was approved by Tohoku University animal use and care committee. All investigations were conducted according to the principles of the Declaration of Helsinki. LPS (10–25 µg/g body weight) (Escherichia coli 0111:B4; Sigma, St Louis, MO, USA) was injected intraperitoneally into 15–20 week old WT and Pin1^−/−^ mice [10], and they were observed for the next 5 days. Kaplan–Meier survival analysis was performed using Stats direct (http://www.statsdirect.com/, StatsDirect, Cheshire, UK).

### Cell Culture

The mouse macrophage cell line RAW264.7 was kindly gifted from K. Nakata (Niigata University, Japan). RAW264.7 was cultured at a concentration of 1.0×10^6^ cells/ml in DMEM supplemented with 10% (vol/vol) FBS, penicillin (100 units/ml), and streptomycin (100 mg/g /ml). RAW264.7 was pretreated with various concentration of Pin1 specific inhibitor PiB prior to LPS stimulation.

Mouse peritoneal macrophages were prepared from the mice injected intraperitoneally with 2 ml of 5% thioglycollate broth (Becton, Dickinson and Company, Franklin Lakes, NJ, USA). Macrophages were separated according to the method described by Freundlich *et al* [17]. Macrophages were cultured at a concentration of 0.5×10^6^cells/ml in DMEM supplemented with 10% (vol/vol) FBS, penicillin (100 units/ml), and streptomycin (100 mg/g /ml). Macrophages were treated with 1–100 ng/ml LPS.

### Construction of expression plasmids and transient transfection

Full-length of Pin1 was generated by PCR from murine macrophage cDNA synthesized using PrimeScript 1st strand cDNA Synthesis Kit (Takara, Otsu, Japan). Full-length of murine Pin1 was sequenced and cloned into pCMS-EGFP expression vector. The expression vector was transiently transfected into RAW264.7 using Lipofectamine 2000 (Invitrogen, Carlsbad, California 92008, USA) as described previously [18].

### Western blot analysis

Macrophages were lysed in SDS sample buffer (pH 6.8, 50 mM Tris-HCl, 2% SDS, 10% glycerol, 5% β-mercaptoethanol, and 1% bromophenol blue). Pin1 and pSer16 Pin1 were detected using rabbit anti-Pin1 antibody (Calbiochem, San Diego, CA, USA) and rabbit anti-pSer16 Pin1 antibody (Calbiochem, San Diego, CA, USA) respectively, followed by anti-rabbit IgG HRP-linked antibody (Cell Signaling Technology, Beverly, MA, USA). Tubulin, used as a control, was detected with mouse anti-α-tubulin antibody (Sigma, St Louis, MO, USA), followed by goat anti-mouse IgG HRP-linked antibody (Santa Cruz Biotechnology, Santa Cruz, CA, USA). Detection was performed by chemiluminescence (Amersham, Arlington Heights, IL, USA). Bands were visualized using a LAS-3000 Image Analyzer (Fuji Film, Tokyo, Japan). Each experiment was performed independently at least three times, and the results of one representative experiment are shown.

### RT-PCR analysis

Total RNA was extracted from macrophages and purified using an RNA Isolation Kit (GE Healthcare, Little Chalfont, Buckinghamshire, UK), and cDNA was synthesized using PrimeScript 1st strand cDNA Synthesis Kit (Takara, Otsu, Japan). RT-PCR analysis of TNFα, IL-6, and TLR4, using β-actin as a control, was performed using the primers shown in Table S2. Quantitative real-time PCR analysis was performed using SYBR Premix Ex Taq (Takara, Otsu, Japan), and the mRNA level was measured using an Applied Biosystems 7300 Real-time PCR system (Applied Biosystems, Foster City, CA, USA). The mRNA levels were normalized to those of β-actin and then normalized to the relative mRNA levels of control samples in each experiment. Results are shown as means±SEM for 3 independent sets of experiments.

### Hematoxylin and eosin staining

After 50–100 h of LPS injection, mice were sacrificed and their tissues were embedded in paraffin. For histological analysis, paraffin-embedded sections were stained with hematoxylin and eosin (Wako, Osaka, Japan).

### Statistical analysis

Values are reported as means ± SEM. The statistical significance of differences between mean values was determined by Student's *t* test. A value of *p*<0.05 was considered statistically significant.

### Pull down assay of PU.1 by Pin1

The pull down assay was performed as written in the previous paper (18). The human macrophage cell line THP-1 and COS-7 cells were cultured at a concentration of 1.0×10^6^ cells/ml. THP-1 cells that were treated with or without 10 ng/ml PMA (12-*O*-Tetradecanoylphorbol 13-acetate) were lysed with Lysis buffer (50 mM Tris-HCl(pH7.5), 150 mM NaCl, 1% NP40, 5 mM EDTA, 1 mM PMSF, 2 µg/ml Aprotinin, 50 mM NaF, 25 mM β-glycerophosphate, 1mM Na_3_VO_4_) . Wild-type Pin1, Pin1 in which Trp34 at the WW domain was mutated to Ala34 (Pin1_W34A_), and Pin1 in which Arg68 and Arg69 at the PPIase domain mutated to Ala68 and Ala69 (Pin1_R68/69A_) were produced as N-terminal glutathione-S-transferase (GST) fusion proteins and bound to the glutathione-Sepharose. These beads were incubated with the cell lysates treated with phosphatase previously or not. In order to determine the Pin1 binding sites of PU.1 in which Thr92 or Ser119 were mutated to Ala were used instead of wild type Pin1. COS-7 cells were used for this assay.

### Immunofluorescent Cell Staining

COS7 cells were transfected with *HA-Pin1* and/or *Flag-PU.1* expression vectors. After 48 h, cells were fixed with 4% paraformaldehyde, treated with HA probe and FLAG M2 antibodies and Alexa Fluor 488- and 595- conjugated secondary antibodies, and observed under fluorescence microscope (Biozero 8000, KEYENCE, Japan).

## Supporting Information

Table S1 Averaged survival time of LPS-injected WT and Pin1-null mice.
(0.03 MB DOC)

Table S2 Primers for real time PCR analysis.
(0.03 MB DOC)
