# Supplementary material for: Prolyl Isomerase Pin1 Protects Mice from Endotoxin Shock
Source: PLoS One. 2011 Feb 4;6(2):e14656. doi: 10.1371/journal.pone.0014656 (PMC3033895; doi:10.1371/journal.pone.0014656)
Supplement: Table S1 — Averaged survival time of LPS-injected WT and Pin1-null mice. (0.03 MB DOC) [file pone.0014656.s001.doc]

TableS1 Averaged survival time of LPS-injected WT and Pin1-null mice

| Genotype | Amount of LPS injection (µg/g) | Death at 100h | Averaged survival time (h) |
| --- | --- | --- | --- |
| WT | 0 | 0/5 | 100 |
|  | 10 | 2/5 | 8.67 ± 0.84 |
| Pin1^-/-^ | 0 | 0/5 | 100 |
|  | 10 | 4/5 | 59.8 ± 12.42 * |
