# Supplementary material for: Prolyl Isomerase Pin1 Protects Mice from Endotoxin Shock
Source: PLoS One. 2011 Feb 4;6(2):e14656. doi: 10.1371/journal.pone.0014656 (PMC3033895; doi:10.1371/journal.pone.0014656)
Supplement: Table S2 — Primers for real time PCR analysis. (0.03 MB DOC) [file pone.0014656.s002.doc]

Supporting Information

TableS2 Primers for real time PCR analysis

| name | primer | sequence | bp | product size (bp) |
| --- | --- | --- | --- | --- |
| TNFα | forward | ctcacactcagatcatcttctc | 25 | 274 |
|  | reverse | ctttctcctggtatgagatagc | 25 |  |
| IL-6 | forward | ttccatccagttgccttcttg | 21 | 108 |
|  | reverse | aggtctgttgggagtggtatc | 21 |  |
| TLR4 | forward | attcagagcggttggtgtatc | 21 | 242 |
|  | reverse | ttcgaggcttttccatccaatagg | 24 |  |
| βactin | forward | tggaatcctgtggcatccatgaaac | 25 | 350 |
|  | reverse | taaaacgcagctcagtaacagtccg | 25 |  |
